# A miniature Ordovician hurdiid from Wales demonstrates the adaptability of Radiodonta

**DOI:** 10.1098/rsos.200459

**Published:** 2020-06-03

**Authors:** Stephen Pates, Joseph P. Botting, Lucy M. E. McCobb, Lucy A. Muir

**Affiliations:** 1 Museum of Comparative Zoology and Department of Organismic and Evolutionary Biology, Harvard University, Oxford Street, Boston, MA 02138, USA; 2 Department of Natural Sciences, Amgueddfa Cymru-National Museum Wales, Cathays Park, Cardiff CF10 3NP, UK; 3 Nanjing Institute of Geology and Palaeontology, Chinese Academy of Sciences, 39 East Beijing Road, Nanjing 210008, People's Republic of China

**Keywords:** Afon Gam Biota, Dol-cyn-Afon Formation, Radiodonta, Hurdiidae, Ordovician, Lagerstätten

## Abstract

Originally considered as large, solely Cambrian apex predators, Radiodonta—a clade of stem-group euarthropods including *Anomalocaris—*now comprises a diverse group of predators, sediment sifters and filter feeders. These animals are only known from deposits preserving non-biomineralized material, with radiodonts often the first and/or only taxa known from such deposits. Despite the widespread and diverse nature of the group, only a handful of radiodonts are known from post-Cambrian deposits, and all originate from deposits or localities rich in other total-group euarthropods. In this contribution, we describe the first radiodont from the UK, an isolated hurdiid frontal appendage from the Tremadocian (Lower Ordovician) Dol-cyn-Afon Formation, Wales, UK. This finding is unusual in two major aspects: firstly, the appendage (1.8 mm in size) is less than half the size of the next smallest radiodont frontal appendage known, and probably belonged to an animal between 6 and 15 mm in length; secondly, it was discovered in the sponge-dominated Afon Gam Biota, one of only a handful of non-biomineralized total-group euarthropods known from this deposit. This Welsh hurdiid breaks new ground for Radiodonta in terms of both its small size and sponge-dominated habitat. This occurrence demonstrates the adaptability of the group in response to the partitioning of ecosystems and environments in the late Cambrian and Early Ordovician world.

## 1. Introduction

Lower Palaeozoic strata known for the exceptional preservation of lightly sclerotized or soft-bodied faunas (*Konservat*-*Lagerstätten*), deposited during the Cambrian and Early Ordovician (540–480 Ma), provide crucial information on the phylogenetic, morphological and ecological diversity patterns of early animals in marine environments (e.g. [1–4]). Radiodonts, a group which includes the large apex predator *Anomalocaris*, are among the best-known animals from these early ecosystems [5–13]. This diverse group of stem-group euarthropods (*sensu* [14]) is known from deposits ranging from China (e.g. [15–20]) to North America (e.g. [7,12,13,21–29]), Europe [25,30,31], North Africa [32,33] and Australia [34–36], reflecting the widespread nature of these (mostly) nektonic animals across different palaeocontinents from the equator to the poles.

**Figure 1.**
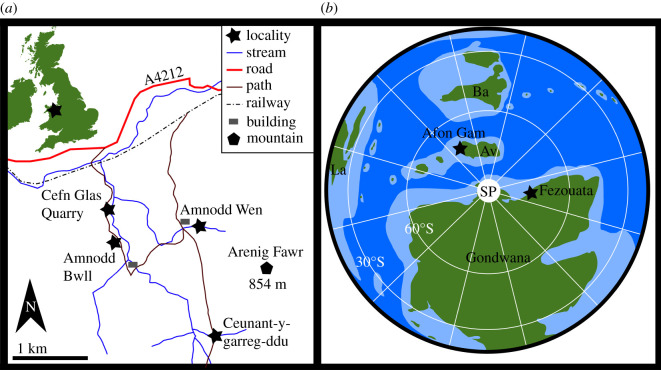
Geographical (*a*) and palaeogeographical (*b*) location of the Afon Gam Biota, Wales, UK. (*a*) Redrawn from [49]; (*b*) constructed using GPlates [50]. Av, Avalonia; Ba, Baltica; La, Laurentia; SP, South Pole.

On account of their lightly sclerotized frontal appendages, head sclerites and mouthparts, which sit anterior to a segmented body with lateral swimming flaps, radiodonts are often among the first animals described from *Konservat-Lagerstätten* (e.g. [8,32,37,38]). They are identifiable to the family, genus or even species level from their frontal feeding appendages alone (e.g. [24,25,29]). Members of the families Amplectobeluidae and Anomalocarididae possess appendages in which endites are alternately long and short on adjacent podomeres (e.g. [11,17]), in contrast with members of Tamisiocarididae and Hurdiidae, in which endites do not alternate in length (e.g. [26,27,31,35]). Tamisiocaridids differ from hurdiids in possessing paired slender endites along the entire appendage, whereas hurdiids have elongate endites (usually broad and recurved) on the five podomeres following the shaft region [24: fig. 1]. In addition, hurdiids sometimes have one to three podomeres with reduced endites distal to the five large-endite-bearing podomeres, and the distal podomeres reduce substantially in size compared with the tall rectangular podomeres in the proximal region [24,26–29].

Recent discoveries have shown that radiodonts occupied an array of ecological niches, from apex predators such as *Amplectobelua*, *Anomalocaris* and *Lyrarapax* [7,11,16–18,20] to sediment sifters such as *Cambroraster*, *Hurdia* and *Stanleycaris* [21,26–28], to the filter feeders *Aegirocassis*, *Pahvantia* and *Tamisiocaris* [31,33,39]. The Cambrian sites from which radiodonts have been reported all possess a rich co-occurring euarthropod fauna (e.g. [8,40–48]).

Here, we report a sub-centimetre-sized radiodont from the Early Ordovician (Tremadocian, *ca* 480 Ma) Afon Gam Biota of the Dol-cyn-Afon Formation, Wales, UK [49]. This is the first representative of this group of stem-group euarthropods from the UK, the first from the palaeocontinent Avalonia and the first from an environment dominated by sponges, rather than euarthropods. The small size of the specimen, which is less than half the size of the next smallest radiodont frontal appendage discovered, and the composition of the co-occurring fauna demonstrate the ecological adaptability of this important Palaeozoic group.

## 2. Material and methods

The radiodont is known from a single specimen, part and counterpart (NMW 2012.36G.90a,b) held at the National Museum Wales (NMW), Cardiff, Wales, UK, under accession NMW 2012.36G. This specimen was collected during fieldwork conducted in 2012, from a loose block in a small quarry at the foot of the Ceunant-y-garreg-ddu stream section (figure 1*a*).

Photographs were taken of the fossil both dry and wet, using a Leica Z6 microscope attached to a Canon 80D camera and stacked using Helicon Focus software.

Palaeogeography for the Tremadocian was reconstructed using GPlates 2.20 [50]. Figures and line drawings were created using Inkscape 0.92.

## 3. The Afon Gam Biota and comparable *Lagerstätten*

The Afon Gam Biota is located in North Wales (figure 1*a*). This deposit was part of the palaeocontinent Avalonia, which was separated from Gondwana towards the end of the Cambrian Period due to the opening of the Rheic Ocean [51]. During the Early Ordovician, Avalonia was located at mid to high latitude in the Southern Hemisphere. Different reconstructions place the continent around 60° latitude (e.g. [52–54]; figure 1*b*), or between 30 and 60° latitude (e.g. [55]).

In common with many other Palaeozoic marine *Konservat-Lagerstätten*, the Afon Gam Biota is rich in sponges. Unusually, trilobites are relatively scarce and other euarthropods are rare, especially when the potential for additional fossil material produced through the act of moulting is considered [49,56]. Given the diversity and preservation of other faunal elements, including sponges, worms and algae, the low density of euarthropods and their overall small size is probably not caused by taphonomic factors [49]. Sub-centimetre-sized bivalved euarthropod carapaces co-occur with rare, multi-centimetre fragments of larger euarthropods, and other taxa such as a several-centimetre priapulid worm, small palaeoscolecids and large agglutinated tubes and sponges [49,57].

The Afon Gam assemblage may usefully be contrasted with the slightly younger Fezouata Biota of Morocco (e.g. [58–61]) and faunas described from Cambrian Burgess Shale-type (BST) deposits (e.g. [46,48]). The community of the Afon Gam Biota is known from a few localities over a relatively small area (figure 1; [49]); however, the community composition does not greatly vary among these sites [49]. The Afon Gam Biota represents a BST community in which euarthropods are a relatively minor part of the total assemblage [49]. By contrast, total-group euarthropods comprise between one-quarter and half of the biodiversity of Cambrian exceptionally preserved faunas (e.g. [45,48,62]). Sponge communities in Cambrian exceptionally preserved faunas are remarkably consistent both temporally and geographically [63], and across all phyla at the genus level, communities became globally more homogeneous across the Stage 4–Wuliuan boundary [46]. Similarly, the Ordovician Fezouata Biota as a whole contains a high diversity of euarthropods sampled from a large number of sites; however, many assemblages are low diversity and dominated by one or two taxa, and some taxa are known only from a handful of specimens at a single site [58,64].

Some younger Ordovician *Konservat-Lagerstätten* exhibit a sponge-dominated ecology superficially similar to the Afon Gam Biota, with a comparable rarity of non-trilobite euarthropods [65,66]. These communities are all faunally distinct from each other, consistent with the increasing community disparity of the Great Ordovician Biodiversification Event. At least among euarthropods, this increasing disparity was either linked with or subsequent to a global restructuring of communities in the late Cambrian marked by several extinction events (e.g. [43,67,68]).

## 4. Fossil description

### 4.1. Terminology and organization of hurdiid frontal appendages

**Figure 2.**
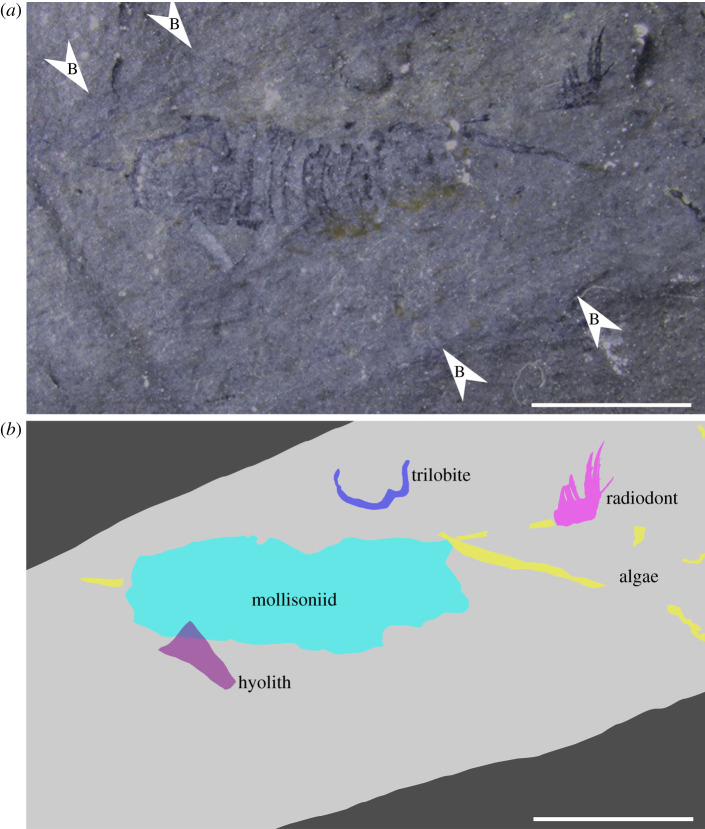
NMW 2012.36G.90a from the Afon Gam Biota, Dol-cyn-Afon Formation, Wales, UK. Accumulation of fossil material in burrow, including hurdiid radiodont frontal appendage. (*a*) Photograph of specimen, taken under water; (*b*) interpretative drawing. B, edge of burrow. Scale bars: 5 mm.

**Figure 3.**
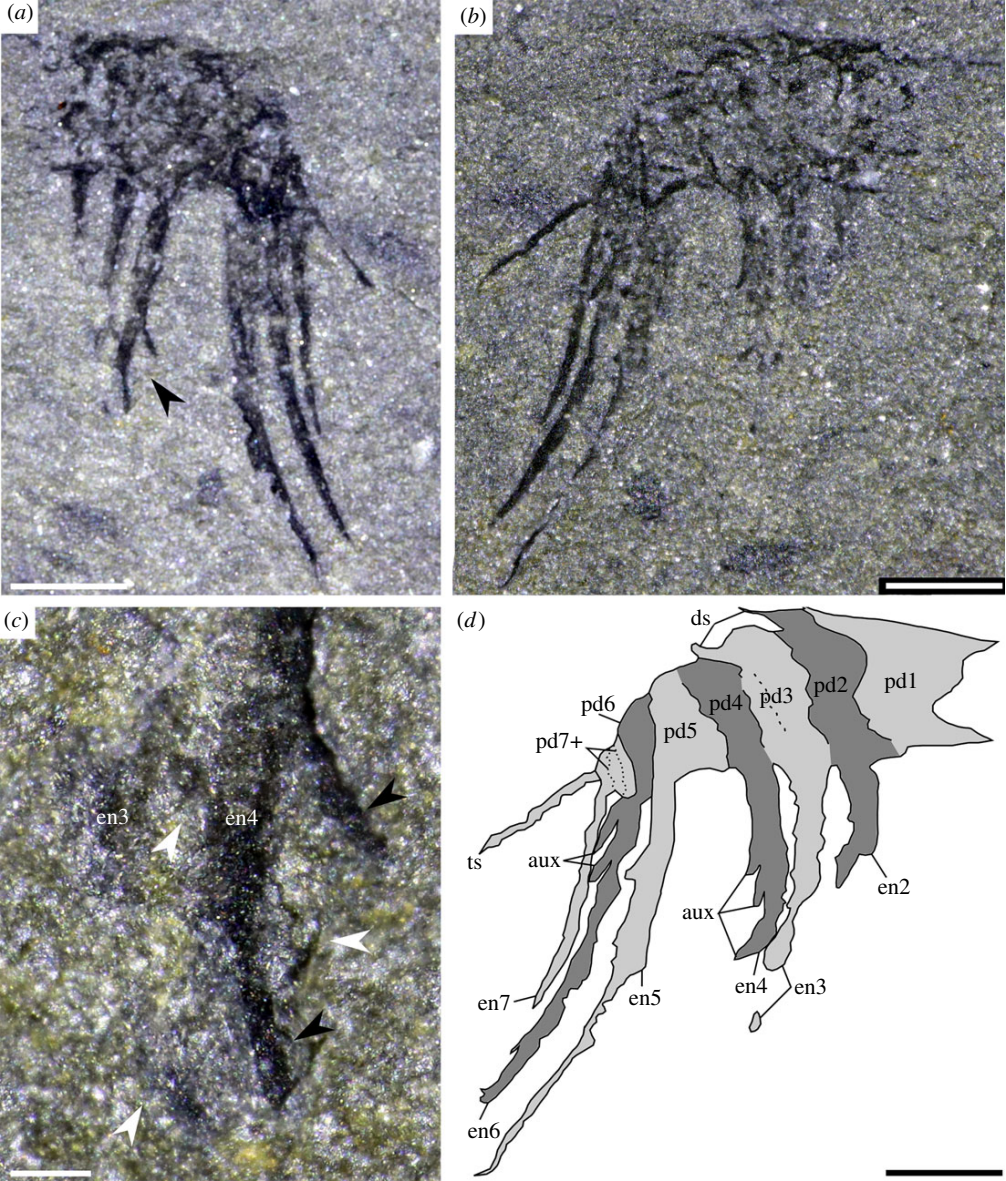
Close-up of hurdiid frontal appendage from figure 2. (*a*) Counterpart NMW 2012.36G.90b; (*b*) part NMW 2012.36G.90a; (*c*) area indicated by black arrow in (*a*), white arrows indicate auxiliary spines on endite 3, black arrows indicated auxiliary spines on endite 4; (*d*) interpretative drawing of the part. aux, auxiliary spines; ds, dorsal spines; en, endite; pd, podomere; ts, terminal spine. Scale bars: (*a*,*b*,*d*) 0.5 mm; (*c*) 0.1 mm.

The single specimen preserved as part and counterpart (NMW 2012.36G.90a, b; figures 2 and 3) probably represents an isolated frontal appendage of a new genus and species of hurdiid radiodont (§4.3). As a result, the description uses terminology associated with hurdiid radiodonts, although alternative taxonomic hypotheses for the specimen are also considered (§4.4).

**Figure 4.**
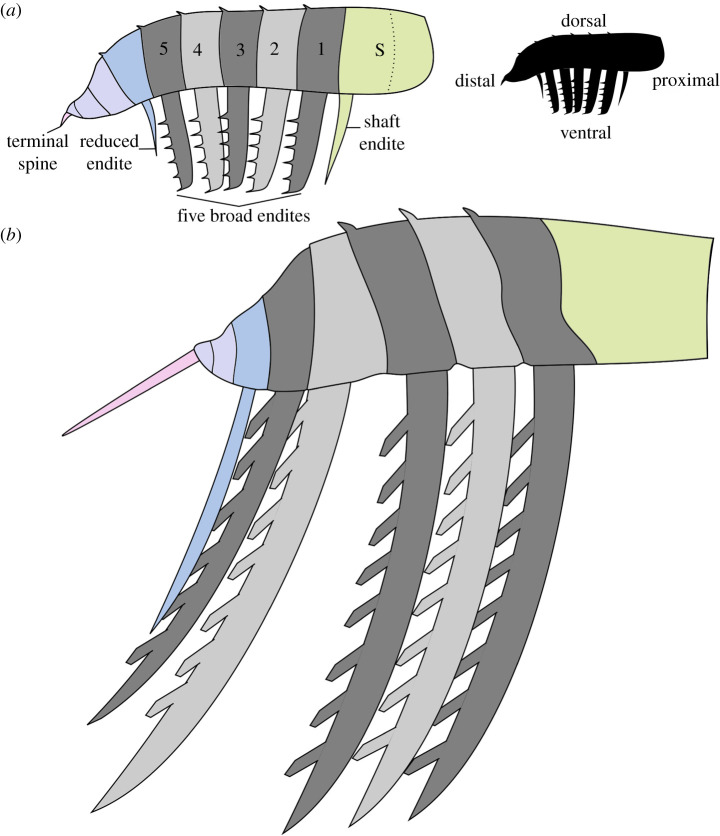
Comparison of an idealized hurdiid appendage and black silhouette illustrating terminology of distal, proximal, dorsal and ventral (*a*) with a reconstruction of the Afon Gam hurdiid (*b*). Colours indicate the proposed homologous parts of the appendage, as labelled in (*a*). 1–5, five podomeres bearing broad recurved endites; S, shaft region.

Radiodont frontal appendages are separated into a ‘shaft’ region, which is generally the poorest known part of the appendage as it is less sclerotized than the endite-bearing ‘distal articulated region’ which follows it [19]. The shaft region (green, figure 4*a*) sometimes bears an endite at its most distal point, though not in all cases (for example, the hurdiid *Peytoia nathorsti* lacks a shaft endite [7,24]).

In hurdiids, the distal articulated region can be separated into two further parts. The proximal five podomeres following the shaft bear elongate endites (grey, figure 4*a*), which are generally unpaired and broad rather than spiniform (a single exception is *Ursulinacaris grallae*, which bears paired slender endites [24]). Endites on these proximal five podomeres in the distal articulated region bear auxiliary spines (rarely setae [33,39]) on their distal margin only. Distal to these five podomeres is sometimes one to three podomeres bearing a reduced endite (blue region, figure 4*a*), although some hurdiids (e.g. *Aegirocassis benmoulai* [33]) do not bear any reduced endites. Sometimes a podomere which does not bear an endite separates the reduced endite-bearing podomere from the five podomeres bearing large endites [26]. Hurdiid appendages terminate in a number of podomeres that do not bear endites, and at least one terminal spine (pink, figure 4*a*).

### 4.2. Secondary concentration of material within a burrow

The specimen NMW 2012.36G.90 is preserved close to the body of a mollisoniid-like animal. As non-trilobite euarthropods are rare in the Afon Gam Biota [49], it might be assumed that the appendage belongs with that body. However, the mollisoniid-like animal and the appendage are inside a burrow, and co-occur with an adjacent hyolith conch, probable algal strands and trilobite fragments (figure 2). This assemblage indicates secondary concentration of the material, and so the two non-trilobite euarthropod specimens are considered to belong to different animals, as is implied by their morphology. It should also be noted that the close association of a hurdiid radiodont appendage with an unrelated body has previously been reported from Cambrian strata of the Holy Cross Mountains, Poland [30], although the context of that association (e.g. whether that material also was concentrated in a burrow) is unknown, as the material comes from a core section.

### 4.3. Description as a hurdiid radiodont frontal appendage

**Figure 5.**
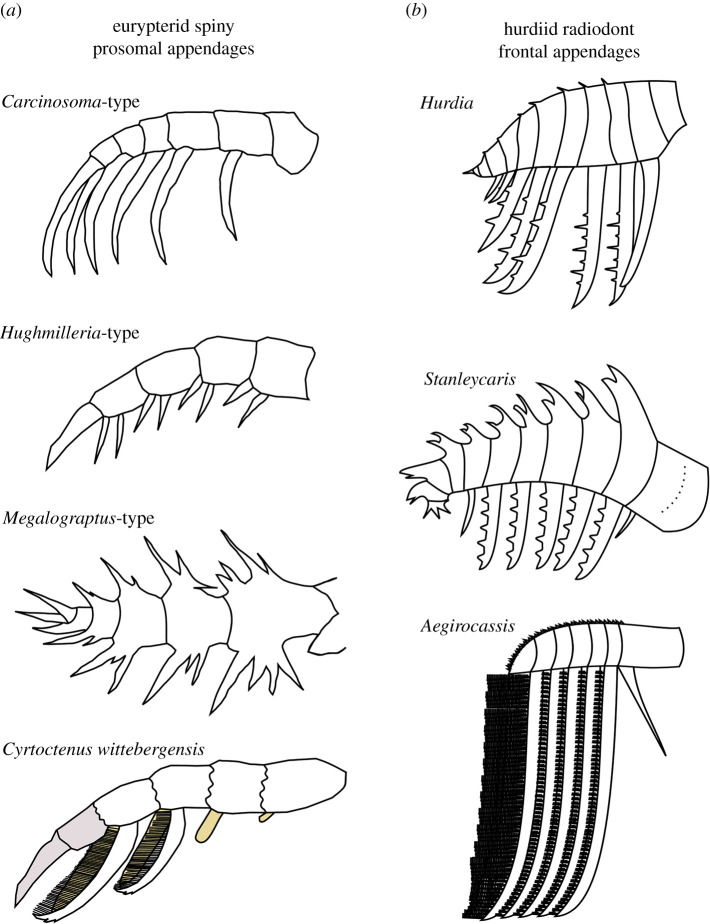
Comparison of distal regions of eurypterid spiniferous prosomal appendages (*a*) and hurdiid radiodont frontal appendages (*b*). Appendages orientated to provide best possible comparison with NMW 2012.36G.90. Reconstructions of *Carcinosoma-*type (taxa such as the Ordovocian *Orcanopterus manitoulinensis*), *Hughmilleria-*type (taxa such as the Ordovician *Paraeurypterus anatolensis*) and *Megalograptus-*type (taxa such as the Ordovician *Pentecopterus decorahensis*) eurypterid spiny appendages redrawn from [69: fig. 9]. Reproduced with permission. *Cyrtoctenus wittebergensis* reconstruction created using data from [70]. Note the finger-like movable spine visible (yellow) underneath the filamentous structures for *Cyrtoctenus wittebergensis*, and comparable structures on two podomeres which do not bear combs proximally*.* The distalmost two podomeres (seven and eight; brown) are not known in this species, and so reconstruction of this part is speculative.

The isolated radiodont frontal appendage, preserved in lateral view, measures 1.8 mm along the dorsal margin from the proximal-most preserved part of the shaft region (figure 3, pd1) to the base of the elongate and straight terminal spine (figure 3, ts), which measures 0.6 mm. Boundaries between six podomeres (figure 3, pd1–pd6) are clearly visible in the part (figure 3*b*,*d*). The preserved width of these podomeres, which represent the shaft (figure 3, pd1) and proximal five podomeres in the distal articulated region (pd2–pd6), is approximately 0.2 mm, with the measured heights reducing from 0.6 mm (figure 3, pd1–pd4) to 0.3 mm (figure 3, pd6). Distally, podomere boundaries are less discernible, but at least three, less than 0.15 mm in height, are visible in this area (figure 3, pd7+). Dorsal spines project from the distal margin of tall rectangular podomeres, most visible on pd2 and pd3 (figure 3, ds). Six blade-like endites are present on the appendage. No endite, either partial or complete, is visible on the shaft podomere. Three partial endites (figure 3, en2–en4) and two complete recurved endites (figure 3, en5 and en6) are present on the proximal five podomeres in the distal articulated region, and one complete but slightly shorter endite (1 mm length) is present distally (figure 3, en7). Complete endites are around four times the length of the podomeres to which they attach; for example, the longest, en5, measures 1.6 mm, and attaches to a podomere measuring 0.4 mm from ventral to dorsal margin. Incomplete partial endites (en2–en4) are expected to have reached similar lengths to the complete endites (en5 and en6) based on comparisons with other hurdiid frontal appendages, and their similar width at the preserved base (figures 4 and 5). Endites bear thin needle-like auxiliary spines which project at an angle between 130 and 160° to the distal margin of the endite to which they attach. Auxiliary spines measure 0.2 mm along their long axis, and are best preserved on the part of the endite closest to the podomere for en4–en6 (figure 3*c*, white and black arrows; figure 3*d*, aux). The distance between the best preserved auxiliary spines (on en4; figure 3*a*, black arrow) is under 0.2 mm (measured between two spines indicated with black arrows in figure 3*c*). There is no evidence that the auxiliary spines were present along the whole length of the endites; indeed, the distal margin of the tipward region appears to be smooth for en5 and en6 (figure 3*a*). However, a comparison of the proximal region of en4, where the auxiliary spine closest to where the podomere meets the endite is only visible in the part not the counterpart (figure 3*b* not figure 3*a*), implies that auxiliary spines may have been present along most of the endite as seen in other hurdiids, and that the lack of auxiliary spines along most of the endites is preservational. It remains possible that spines are only found on the part of the endite closest to the podomere.

The presence of a blade-like endite on each of the proximal five podomeres in the distal articulated region, tall rectangular podomeres that reduce in height markedly in the distal portion and the presence of auxiliary spines only on the distal margin of endites are all characters which support the identification of this specimen as the frontal appendage of a hurdiid radiodont. Under this hypothesis, the Welsh radiodont would lack a shaft endite, just as in the hurdiid *Peytoia nathorsti,* and the five proximal endites would be equivalent to the five blade-like endites known in all other hurdiids except for *U. grallae,* in which endites on these five podomeres are slender and paired [24] (grey podomeres and endites in figure 4). The distalmost endite (en7) would be equivalent to the reduced distal endites (blue endite in figure 4; ‘den’ in fig. 2 of [24]; ‘enditic spines’ of [26]) known in *Cambroraster*, *Hurdia, Stanleycaris, Ursulinacaris* and a taxon not yet formally described from the Burgess Shale (?*Laggania* of [29]), although such a comparatively long distal endite is only known in the latter two hurdiid radiodonts [21,23,24,26,27,29].

The orientation of the auxiliary spines (slightly oblique and pointing towards the tip of the endite, rather than perpendicular to the endite) is unique among hurdiids, but has been observed in the other three radiodont families: amplectobeluids (e.g. *Amplectobelua symbrachiata*; [10]); anomalocaridids (e.g. *Anomalocaris canadensis*; [11]); and tamisiocaridids (e.g. *Tamisiocaris borealis;* [31]). In each of these cases, auxiliary spines project from both the distal and proximal margins of endites. In all phylogenetic analyses which have attempted to shed light on the internal relationships of radiodonts, the sister group to hurdiids has been resolved as tamisiocaridids (‘Cetiocaridae’ of [31]; [15,20,31,33,39]) or in one case, amplectobeluids [26]. The presence of this character in a hurdiid is, therefore, less surprising, given its appearance in the sister group of the family, and in fact all other radiodont families. Indeed, given the phylogenetic frameworks of all these studies, the case observed in the Welsh radiodont can be considered to be the ancestral condition for Radiodonta, with the case in other hurdiids (a row of spines perpendicular to the endite) derived.

While the arrangement (five blade-like endites proximal to a reduced endite) and shape (recurved distally and broad) of endites is broadly similar to many hurdiids such as *Hurdia,* and *Stanleycaris* (figure 5) [21,27,28], the *ca* 4 : 1 ratio of endite length to podomere height in the Welsh hurdiid is only otherwise observed in the filter-feeding nektonic *Aegirocassis* (figure 5) and *Pahvantia*, and the eudemersal *Cambroraster* [26,33,39]. The orientation and spiniform nature of the auxiliary spines, the length and morphology of the terminal spine (a length that far exceeds that observed in other radiodonts, relative to the length of the appendage) and the relative length of the reduced distal endite provide a unique combination of characters that suggest that this specimen represents a new genus and species of hurdiid radiodont (reconstructed in figure 4*b*).

### 4.4. Alternative taxonomic hypotheses

Given the partial nature of the specimen, its small size, unusual depositional setting and novel characters—especially the length of the terminal spine—it is necessary to consider alternative taxonomic hypotheses. In two previous cases, material originally described as a lobopodian has been reidentified as a radiodont frontal appendage [23,25,71–73], and, given the small size of specimen NMW 2012.36G.90, such a hypothesis should be given some consideration. The lobopods of some lobopodians such as *Aysheaia pedunculata* from the Burgess Shale taper to a clawed tip. When preserved laterally compressed and curved towards the head end, these structures can give an overall blade-like appearance, similar to hurdiids [23: fig. 3D]. However, a lobopodian affinity for this specimen is discounted by the presence of boundaries (interpreted here as podomere boundaries) which cross the entire specimen and are more widely spaced than would be expected for the annulated body of a lobopodian.

The presence of podomere boundaries allows the identification of this specimen as a total-group euarthropod appendage. Eurypterids, crown-group euarthropods (Chelicerata) also known from the early Palaeozoic, possessed non-biomineralized appendages, some of which bear a superficial resemblance to specimen NMW 2012.36G.90. Eurypterids bear six pairs of legs under the prosoma, which are referred to as appendages I–VI from anterior to posterior. Appendage I bore the chelicerae, while appendages II–V were walking legs with gnathobases, and sometimes spines [69,74]. In stylonurine eurypterids, appendages II–VI may differ slightly in size but all display broadly the same morphology, whereas in the other branch of the eurypterid tree, in eurypterines, appendages II and III have a raptorial function, appendages IV and V are walking legs and appendage VI is a specialized swimming paddle [69,74: fig. 26]. The spiniferous walking legs bear the most similarity to hurdiid radiodont frontal appendages. Eleven spiny walking leg morphologies were identified by Tollerton [69: fig. 9], and below we consider first morphologies known from the Ordovician, and then secondly a morphology known in derived freshwater eurypterids with recurved comb structures similar to the endites of hurdiid radiodont frontal appendages (figure 5).

The oldest known eurypterid, *Pentecopterus decorahensis*, was described from the Winneshiek Lagerstätte (Darriwilian; Iowa, USA [74]). This animal is younger than NMW 2012.36G.90, and so a eurypterid affinity for the Welsh animal would make it the oldest member of this group. *Pentecopterus* is a member of the family Megalograptidae, with other Ordovician eurypterids assigned to this family and Onychopterellidae, Orcanopteridae and Rhenopteridae [74–77]. A final Ordovician eurypterid, *Paraeurypterus anatolensis* from Turkey, has not been assigned to a family but belongs to the eurypterine branch (rather than the stylonurine branch) [78]. Of these groups, megalograptids possessed *Megalograptus-*type legs (figure 5) [69], onychopterellids and *P. anatolensis* bore *Hughmilleria-*type legs (figure 5) [69,75,78] and rhenopterids had unspiniferous legs [69]. Non-spiniferous, *Hughmilleria-*type and *Megalograptus-*type eurypterid walking legs share very few morphological characters with specimen NMW 2012.36G.90 beyond the presence of podomere boundaries, and spines in the latter two. The morphology of the spines, number per podomere and orientation, as well as position on the podomere, all differ from what is seen in the Welsh specimen, even when the leg is orientated to be as similar as possible (figure 5). For example, some elongate spines are known on some appendages of *P. decorahensis*; however, these are either straight or curve the opposite direction to the Afon Gam appendage, and are on the same podomeres as other spines [74: figs 3 and 10D]. Of the Ordovician eurypterids, the orcanopterid *Orcanopterus manitoulinensis* (Upper Ordovician; Manitoba, Canada) with its *Carcinosoma-*type legs (figure 5) [69,76] is most similar to the specimen described in this study. Although incomplete, the prosomal legs II–V of the Canadian eurypterid possess a single recurved spine from a single podomere; however, these spines curve in the opposite direction relative to the appendage than the endites in the Welsh animal (and hurdiid endites in general; figures 4*a* and 5) [76: figs 3.4 and 4.3], a character shared with other *Carcinosoma-*type appendages [69]. Distal podomeres in *O. manitoulinensis* bear only one spine, even on appendage V, where four podomeres can be observed [76: fig. 4.3]. This morphology contrasts with the multiple podomeres that bear endites in the Welsh animal, although it should be noted that other *Carcinosoma-*type appendages do preserve multiple podomeres bearing curved spines (figure 5). In addition to differences in the spine morphology in *Carcinosoma*-type appendages to the endites observed in the Welsh animal, the podomeres are also a different shape. In the Manitoban eurypterid, they are square to elongate rectangles, in contrast with the tall and thin rectangles in the Afon Gam appendage.

Disregarding temporal or environmental constraints, the comb-bearing appendages of the sweep-feeding eurypterid genus *Cyrtoctenus* (family Hibbertopteridae) share a number of morphological characters with the Afon Gam specimen, and hurdiid appendages in general [28,70,79]. This stylonurine family is known from Devonian and younger deposits, exclusively from freshwater environments, and has been considered a highly specialized and unusual group of eurypterids [80]. *Cyrtoctenus* appendages bear elongate recurved combs with numerous (more than 80) finely spaced filament structures extending from the concave margin (figure 5). The shape of the comb and margin bearing auxiliary structures is consistent with what can be observed in NMW 2012.36G.90; however, there are a number of significant morphological differences (figures 3–5). In *Cyrtoctenus*, each comb is associated with a movable finger-like spine (visible underneath filaments in figure 5) [70,81], of which there is no evidence in the Welsh specimen. In addition, although the appendages of *Cyrtoctenus* are not completely known, in the South African taxon *C. wittebergensis*, combs are present on at least two, and potentially up to four podomeres—combs are absent on the proximal four podomeres, present on podomeres five and six while podomeres seven and eight are not preserved [70]. Podomere seven could only have hosted a very small comb and it was almost certainly absent on the distalmost podomere eight, due to the physical constraints of hosting these combs on a walking leg [70]. In the type species, *C. peachi*, each comb and movable finger-like spine was originally described as a separate abdominal appendage, with five (appendages A through E) described altogether [79]. Comparing with what is known in *C. wittebergensis,* movable spines (appendages C, D and E) and one comb (appendage A) can be recognized in the type species, while appendage B can be interpreted either as a modified comb or movable spine [70]. Even assuming the maximum possible number of combs in *Cyrtoctenus* (four, extrapolated from the maximum number of movable fingers in *C. peachi* or the number of observed combs in *C. wittebergensis* plus the number of podomeres not preserved), an additional two are present in the Welsh animal. The combs differ in relative size in *Cyrtoctenus* and the Afon Gam animal. In *C. wittebergensis*, the distalmost comb on podomere six was probably longer than that of podomere five, as implied by the relative size of the accompanying movable finger [70], the opposite case to what is seen in NMW 2012.36G.90, in which the distalmost endite is reduced—a morphology also observed in other hurdiids, as mentioned above.

Thus, despite some morphological similarities between both lobopodian bodies and eurypterid spiny legs and the Welsh animal, including the presence of broad curved spines, some of which bear slender projections on the concave margin, many more characters support the interpretation of specimen NMW 2012.36G.90 as the isolated frontal appendage of a hurdiid radiodont. Of the two characters unknown in other hurdiid radiodonts—the elongate terminal spine and auxiliary spines orientated towards the tip of endites—the second is known in all other radiodont families and probably represents the plesiomorphic state for Radiodonta. The elongate terminal spine (relative to appendage length) is unknown in other members of the order, and could reflect ecological specialization. This character may also have changed during the growth of the animal, becoming shorter relative to appendage length; however, our current knowledge of radiodont development (discussed briefly below) does not support this hypothesis.

## 5. Discussion

### 5.1. A miniature Ordovician radiodont

The Afon Gam animal represents the smallest known hurdiid, and potentially radiodont, currently known. Literature data for appendage : body length ratios in complete radiodonts have previously been compiled (supplemental note in [39]). Extrapolating from these data and considering additional information from *Cambroraster* [26] and *Hurdia* [28], this animal was probably between 6 and 15 mm in length, although it could conceivably have been as small as *ca* 3.5 mm.

The extrapolated size range of 6–15 mm for this animal based on the size of the frontal appendages comes from comparisons with other hurdiid radiodonts. Complete specimens of the hurdiid *Peytoia nathorsti* from the Burgess Shale possess bodies between three-and-a-half and four times the length of their frontal appendages [7: figs 30 and 31; 39], giving the lower bound estimate for the likely length of this animal, 6 mm. Among radiodonts as a whole, complete specimens of *An. canadensis* can have frontal appendages approximately half the total length of the animal [11: figs 5 and 7; 39], which gives a possible, albeit unlikely, lowest bound estimate of *ca* 3.5 mm for the Welsh animal.

The relative length of appendages to body length is more difficult to determine in *Cambroraster* and *Hurdia*, due to the way that frontal appendages are often obscured by the carapace elements, and/or preserved incomplete and obliquely in complete or slightly disarticulated body specimens [26,28]. For *Hurdia*, a specimen in lateral view [28: fig. 21C,D] best shows the relationship between the length of the body and frontal appendages, as the carapace does not obscure the length of either; however, the proximal part of the appendage is overlain by the oral cone, preventing exact measurement. Taking only the visible portion, the frontal appendage in this specimen is approximately two-sevenths of the length of the body (not including the oral cone or carapace), one-fifth the length of the body including the oral cone but not the carapace and one-sixth of the length of the body including both the oral cone and the carapace. Taking this specimen alone gives a length of *ca* 9–11 mm for the Welsh hurdiid, although the more incomplete appendages in *Hurdia* specimens preserved in dorsal and lateral view indicate that frontal appendages of other specimens may have been shorter relative to body length (e.g. [28: fig. 3]). The comparison that gives the largest estimate for the body of the Welsh hurdiid comes from *Cambroraster.* A single specimen shows a well-preserved appendage adjacent to an isolated central carapace element [26: fig. 1g], and the length along the distal margin of this appendage is approximately one-fifth of the length from the anteriormost point of the carapace to the posteriormost point. The central carapace element in the holotype of *C. falcatus* [26: fig. 1a,b] extends over three-quarters of the total length of the animal. This ratio gives a conservative estimate that the frontal appendage is one-eighth of the total animal length. Extrapolating this for the Welsh hurdiid yields a body length of 15 mm, albeit based on numerous assumptions and approximations. Given the unusual ratio of frontal appendage size to body length in the eudemersal *Cambroraster* compared with other hurdiids, it is likely that the size of the Afon Gam radiodont was towards the lower end of the likely range suggested here (6–15 mm), and it is, therefore, likely that this animal represents the smallest known radiodont, although the significant uncertainties in these extrapolations should be acknowledged. Regardless, the size of the frontal appendage of the Welsh hurdiid is less than half that of the next smallest radiodont known, a 16 mm long juvenile *Lyrarapax* from the Chengjiang Biota with a well-preserved appendage *ca* 4–5 mm long (measured along the dorsal margin) [20]. The very small size of the Welsh hurdiid is consistent with the generally small size of other non-trilobite euarthropods from the Afon Gam Biota [49].

The status of the Welsh specimen as an adult or juvenile cannot be determined beyond doubt from only a single specimen, and indeed, very little is known about radiodont development. The few juveniles identified—the *Lyrarapax* mentioned above [20] and a small specimen of *A. symbrachiata*, also from the Chengjiang Biota [9: fig. 3], which measures *ca* 90 mm in body length (frontal appendages *ca* 20 mm)—show few morphological differences in their frontal appendages to adults of the same species, with no significant changes in the length of the terminal spine. A change in the orientation of auxiliary spines cannot be evaluated for *A. symbrachiata* or *L. trilobus* as this character is not preserved in the juvenile of the former radiodont, and is not known in the juvenile or adult of the latter [9,15,16,20]. The single row of small spines on the hypertrophied endite in *L. unguispinus* does not appear to be different in either the smallest (16 mm body length) specimen [20] or the largest (*ca* 80 mm body length) [15]. It is not known whether juvenile radiodonts were preceded by a larval stage, but from the current data, it would be expected that should the Afon Gam hurdiid specimen represent a juvenile, the adult would display a similar morphology. This consideration strongly implies that both the characters observed in this animal that are not known in other hurdiids—the orientation of the auxiliary spines and the length of the terminal spine relative to appendage length—would be present in adult specimens, regardless of whether the single specimen preserved is an adult or a juvenile.

### 5.2. Adaptability of Radiodonta

This description of a radiodont from the Afon Gam Biota, the first from Avalonia and the UK, adds to the substantial evidence supporting a wide geographical spread for this group in the early Palaeozoic. As the second Ordovician deposit from which a radiodont has been discovered, after the Fezouata Biota [32,33], this occurrence of a hurdiid radiodont on a new palaeocontinent provides a rare data point for understanding the post-Cambrian geographical range and diversity of this clade. Radiodonts are well known from Cambrian *Konservat-Lagerstätten*, on account of the relatively high preservation potential of their lightly sclerotized frontal appendages and oral cones, and their large size, while their ecological adaptability allowed them to exploit infaunal, epifaunal and planktonic food sources (e.g. [20,26–29,31,33,35,39]). However, this report of a centimetre-sized radiodont from an environment not rich in macroscopic, epifaunal food sources or other euarthropods shows a complementary way in which radiodonts were able to adapt to different environments, in this case to one in which the majority of other euarthropods appear to have been unable to thrive.

The Afon Gam Biota was probably a more hostile environment for radiodonts, and euarthropods in general, than the Cambrian BST *Konservat*-*Lagerstätten* from which they are best known, and the near-contemporaneous Fezouata Biota. This is demonstrated by the very different ecological balance in these BST faunas compared with the Ordovician Welsh deposit (e.g. [46,49,58]). The observed faunal composition within the Afon Gam Biota apparently left little for a large macropredator to consume, as the majority of preserved, motile epifaunal animals were biomineralized, in contrast with the wide variety of soft or lightly sclerotized motile epifaunal prey known from Cambrian BSTs and the Fezouata Biota. This is emphasized by the lack of large euarthropods known (so far) in the Afon Gam Biota [49], and by the presence of a wide variety of euarthropods of different sizes, including hurdiid radiodonts, in the Fezouata Biota [32,33]. The abundant worm fauna of the Afon Gam Biota, indicated largely by tubes and lined burrows [49,57], appears to have been dominantly infaunal. The question of whether the new radiodont described here shows adaptations to specific environmental differences between the Afon Gam Biota and other radiodont-bearing deposits should be considered in the light of the Ordovician ecological context. A recent detailed stratigraphic study of the early Cambrian Chengjiang biota demonstrated two distinct types of horizon: sponge-dominated background beds and euarthropod-dominated event beds, reflecting deep dysoxic and shallow oxic environments, respectively [82]; some other early Cambrian exceptionally preserved faunas from deep-water settings are also sponge-dominated [83]. However, the Afon Gam trilobite fauna closely resembles that of the shallow-water Sheinton Shales of Shropshire, and sedimentological data suggest that the Dol-cyn-Afon Formation was most likely not deposited in a deep-water setting [49].

Similarly, although some modern demosponges are able to thrive in very low-oxygen conditions (e.g. [84]), that may not be the case for other sponge groups, and there are many modern sponge-dominated communities that are not related to low oxygen levels. Indeed, the factors controlling sponge dominance are diverse; these faunas are typically associated with high nutrient levels (e.g. upwelling zones), but also other factors such as steeper slopes [85]. As sponge-dominated Ordovician assemblages also occur more widely in Wales [65,86], and the Afon Gam Biota includes a diverse range of taxa such as echinoderms and tubiculous worms, and infauna, invoking low oxygenation to explain the ecology of the Afon Gam Biota does not seem justifiable; regional or wider ecological shifts must, therefore, be considered.

Strikingly, the largest radiodont ever discovered, the 2 m long hurdiid *A. benmoulai*, is from the near-contemporaneous Early Ordovician Fezouata Biota [32,33]. This Moroccan giant is two to three orders of magnitude larger than the Welsh hurdiid. In contrast with Cambrian BSTs, which are generally euarthropod-dominated and show remarkable consistency even at the genus level across multiple palaeocontinents over an approximately 25 Myr time span [46,63], Ordovician and younger *Konservat-Lagerstätten* exhibit marked disparities in their biotic compositions. Sponge-dominated sites, such as the Afon Gam Biota, are known from the Ordovician of Wales [63,65,86,87], and also from Cambrian and Ordovician strata of China [66,83], whereas Late Ordovician *Konservat-Lagerstätten* of Canada contain marginal-marine algal- or euarthropod-dominated assemblages [88,89], and the Silurian Kalana Formation preserves an exquisite algal fauna alongside crinoids and other non-euarthropod animals [90–93]. During the Ordovician biodiversification, within-community (α), between-community (β) and inter-provincial (γ) diversity all increased [94], resulting in greater differences between communities (including those preserved in *Konservat-Lagerstätten*) during the Ordovician than in the Cambrian. This pattern is clearly apparent when comparing the Cambrian and Ordovician sponge faunas [63]. In addition, at a global scale, the Ordovician biodiversification involved a pronounced shift towards suspension feeding and a reduction in mobile benthic predators (e.g. [94,95]). The rise of large nektonic predators such as orthocone nautiloids and eurypterids limited the role for radiodonts as apex predators during the Ordovician, and the convergent evolution of jaws in the Silurian increased the competition for this ecospace yet further [96,97]. The exploration of a vast range in body sizes within Ordovician radiodonts may reflect adaptations to the early stages of these global ecological changes.

For the specific case of the Afon Gam Biota, the small size of the hurdiid radiodont may reflect an adaptation to the small size and low abundance of epifaunal motile non-biomineralized prey in the environment.

## 6. Conclusion

The description of a miniature hurdiid from an Ordovician *Konservat-Lagerstätte* in Wales is the first report of a radiodont from palaeocontinent Avalonia and the modern-day UK. This animal has the smallest known frontal appendages of any member of Radiodonta. An extrapolation of its body size from this small frontal appendage suggests that this animal is the smallest hurdiid and probably the smallest radiodont ever discovered.

This paper is also the first report of a radiodont from an environment not dominated by euarthropods and highlights the adaptability of the group to a changing Ordovician world. There are greater differences between different Ordovician communities than their Cambrian counterparts, and available ecospace for large nektonic predators was restricted yet further by the emergence of other groups such as orthocone nautiloids. The small size of the Welsh hurdiid is interpreted as one adaptation towards the local ecological conditions of the sponge-dominated Dol-cyn-Afon Formation. Two alternative explanations, that the water depth or oxygenation at Afon Gam sites was significantly different to Cambrian *Konservat-Lagerstatten*, are rejected based on the co-occurring fauna.

## Supplementary Material

Reviewer comments

## Data accessibility

The specimen studied here is accessioned at Amgueddfa Cymru-National Museum Wales under accession NMW 2012.36G. No additional data were generated during the course of the study.
