# Supplementary material for: A miniature Ordovician hurdiid from Wales demonstrates the adaptability of Radiodonta
Source: R Soc Open Sci. 2020 Jun 3;7(6):200459. doi: 10.1098/rsos.200459 (PMC7353989; doi:10.1098/rsos.200459)
Supplement: Reviewer comments [file rsos200459_review_history.pdf]

THE ROYAL SOCIETY
PUBLISHING

ROYAL SOCIETY
OPEN SCIENCE

A miniature Ordovician hurdiid from Wales demonstrates the adaptability of Radiodonta

Stephen Pates, Joseph P. Botting, Lucy M. E. McCobb and Lucy A. Muir

Article citation details
R. Soc. open sci. **7**: 200459.
http://dx.doi.org/10.1098/rsos.200459

Review timeline
Original submission: 25 March 2020
Revised submission: 15 May 2020
Final acceptance: 18 May 2020

Note: Reports are unedited and appear as submitted by the referee. The review history appears in chronological order.

Review History

RSOS-200459.R0 (Original submission)

Review form: Reviewer 1

Is the manuscript scientifically sound in its present form?
Yes

Are the interpretations and conclusions justified by the results?
No

Is the language acceptable?
Yes

Do you have any ethical concerns with this paper?
No

Have you any concerns about statistical analyses in this paper?
No

Recommendation?
Major revision is needed (please make suggestions in comments)

Reports © 2020 The Reviewers; Decision Letters © 2020 The Reviewers and Editors; Responses © 2020 The Reviewers, Editors and Authors. Published by the Royal Society under the terms of the Creative Commons Attribution License http://creativecommons.org/licenses/by/4.0/, which permits unrestricted use, provided the original author and source are credited

Comments to the Author(s)
The subject of this paper is a single small, fragmentary fossil preserved as a black film. It is either a hurdiid as the authors argue or else it is a fragment of some other kind of organism. Unlike Ursalinacaris (or other hurdiids), the inferred ventral spines greatly increase in length along a series of inferred proximal to distal podomeres, being really long on inferred podomeres 5 and 6. This could simply be taxonomic novelty (a new way of being a hurdiid) but it is so fundamentally different from radiodont ventral spine variation that it makes me worry that the specimen could be something else completely. Likewise having a very elongate, straight, needle-like terminal spine is off the scale for hurdiids (or radiodonts more widely). The "dorsal spines" are poorly preserved: if it's a radiodont frontal appendage then that is what they are but on their own they're not a strong argument for this being a radiodont. The "auxiliary spines" seem to be OK (they're on the inferred distal edge of the ventral spines and have consistent orientations among the few preserved on the specimen – although, as the authors note, their obliqueness is unlike hurdiids).

The "Adaptability of Radiodonta" section could be shortened. It comes across as trying to extract as much impact as is possible from a single fragment. The major novel conclusions of the paper (such as a radiodont being much smaller than thought possible, and occurring in a setting where radiodonts don't usually seem to have lived) could also be seen as reasons to wonder if it actually is a radiodont.

I can appreciate that it won't be easy to tease much more out of the specimen, e.g., trying different imaging approaches likely won't make it reveal unambiguous characters. But I'd urge the authors to throw everything possible at it.

Review form: Reviewer 2

Is the manuscript scientifically sound in its present form?
Yes

Are the interpretations and conclusions justified by the results?
No

Is the language acceptable?
Yes

Do you have any ethical concerns with this paper?
No

Have you any concerns about statistical analyses in this paper?
No

Recommendation?
Accept with minor revision (please list in comments)

Comments to the Author(s)
This is a nice account of an interesting new appendage, probably belonging to a radiodont panarthropod. This group is uncommon in post-Cambrian deposits and as such the discovery is of interest for understanding their range and diversity.
I think that the argument that this is a radiodont appendage is reasonably convincing, although it might be strengthened if specific descriptive comparisons were made with other possible identities, for example eurypterids which can sometimes have quite hurdiid-like appendages.

My main concern is that insufficient evidence and argumentation is provided to suggest that this animal was a filter feeder. I find this interpretation unconvincing given the few and very short auxiliary spines visible in the single specimen. Undoubtedly prey size must have been small given the size of the animal, but could it not also have been feeding on small epibenthic or infaunal organisms? I suggest either a more pluralistic discussion of the evidence for and against different feeding types, or to simply acknowledge the uncertainty in interpretation given the limited fossil material.
Specific comments:
45 – "vast" may be an overstatement
134, 175 – Where was this measurement taken? There seem to be very few auxiliary spines and they are sparsely distributed. Are you inferring that some are not visible or not preserved, such that originally there would have been a more continuous array?
160 – Less than 8mm seems like a low estimate for an appendage of length 1.8mm. The reference cited (39) for estimating the size of the complete animal is not suited for this purpose considering that Pahvantia is not known from complete specimens. Refs (7), (26)-(28) include more complete hurdiid specimens for size estimation. Based on a rough estimate from the most complete specimens in these papers, I would think a body size of 10-20mm would be more reasonable. This would make it roughly on par with the small Lyrarapax specimen described in (20) – allowing for some uncertainty in size estimation might warrant tempering claims that this is the smallest known radiodont.
167 – Might be worth emphasizing the caveat that very little is known about early radiodont development (even the smallest juvenile Lyrarapax might be far along in development, if for example it was preceded by a larval stage).
180 – (Also other places where this is discussed in the text) I don't think it's justified to infer convergence without providing a phylogenetic tree in support.
203 – Couldn't the lack of soft/lightly sclerotized organisms be due to taphonomic bias? The general rarity of euarthropods in this biota contrasts with the localized preservation of this radiodont appendage along with a mollisoniid. Could this be the result of atypical local taphonomic conditions?
225 – So you argue that oxygen and water depth are not likely to explain faunal differences. Then you generally invoke regional ecological shifts as an alternative explanation. Is the next section meant to expand on this by proposing that the greater beta diversity in the Ordovician explains the Afon Gam biotic composition? I don't quite see the logic here, as the GOBE may explain the global pattern, but we still lack a cause for the differentiation of the Afon Gam biota from contemporaneous deposits (be that oxygen, water depth or some other factor not considered). In other words, it may fit the global pattern, but doesn't explain the specific case.
230-245 – the discussion in this section reads a bit disjointed from the rest of the text and the connection with the new fossil material is not very evident. It also seems to mix together a few distinct ideas. I recommend to simplify and clarify this section.

Decision letter (RSOS-200459.R0)

We hope you are keeping well at this difficult and unusual time. We continue to value your support of the journal in these challenging circumstances. If Royal Society Open Science can assist you at all, please don't hesitate to let us know at the email address below.

Dear Dr Pates,

The editors assigned to your paper ("A miniature Ordovician hurdiid from Wales demonstrates the adaptability of Radiodonta") have now received comments from reviewers. We would like you to revise your paper in accordance with the referee and Associate Editor suggestions which can be found below (not including confidential reports to the Editor). Please note this decision does not guarantee eventual acceptance.

Please submit a copy of your revised paper before 16-May-2020. Please note that the revision deadline will expire at 00.00am on this date. If we do not hear from you within this time then it will be assumed that the paper has been withdrawn. In exceptional circumstances, extensions may be possible if agreed with the Editorial Office in advance. We do not allow multiple rounds of revision so we urge you to make every effort to fully address all of the comments at this stage. If deemed necessary by the Editors, your manuscript will be sent back to one or more of the original reviewers for assessment. If the original reviewers are not available, we may invite new reviewers.

To revise your manuscript, log into http://mc.manuscriptcentral.com/rsos and enter your Author Centre, where you will find your manuscript title listed under "Manuscripts with Decisions." Under "Actions," click on "Create a Revision." Your manuscript number has been appended to denote a revision. Revise your manuscript and upload a new version through your Author Centre.

When submitting your revised manuscript, you must respond to the comments made by the referees and upload a file "Response to Referees" in "Section 6 - File Upload". Please use this to document how you have responded to the comments, and the adjustments you have made. In order to expedite the processing of the revised manuscript, please be as specific as possible in your response.

In addition to addressing all of the reviewers' and editor's comments please also ensure that your revised manuscript contains the following sections as appropriate before the reference list:

• Ethics statement (if applicable)
If your study uses humans or animals please include details of the ethical approval received, including the name of the committee that granted approval. For human studies please also detail whether informed consent was obtained. For field studies on animals please include details of all permissions, licences and/or approvals granted to carry out the fieldwork.

• Data accessibility
It is a condition of publication that all supporting data are made available either as supplementary information or preferably in a suitable permanent repository. The data accessibility section should state where the article's supporting data can be accessed. This section should also include details, where possible of where to access other relevant research materials such as statistical tools, protocols, software etc can be accessed. If the data have been deposited in an external repository this section should list the database, accession number and link to the DOI for all data from the article that have been made publicly available. Data sets that have been deposited in an external repository and have a DOI should also be appropriately cited in the manuscript and included in the reference list.

If you wish to submit your supporting data or code to Dryad (http://datadryad.org/), or modify your current submission to dryad, please use the following link:
http://datadryad.org/submit?journalID=RSOS&manu=RSOS-200459

• Competing interests
Please declare any financial or non-financial competing interests, or state that you have no competing interests.

• Authors' contributions
All submissions, other than those with a single author, must include an Authors' Contributions section which individually lists the specific contribution of each author. The list of Authors should meet all of the following criteria; 1) substantial contributions to conception and design, or acquisition of data, or analysis and interpretation of data; 2) drafting the article or revising it critically for important intellectual content; and 3) final approval of the version to be published.

All contributors who do not meet all of these criteria should be included in the acknowledgements.

We suggest the following format:
AB carried out the molecular lab work, participated in data analysis, carried out sequence alignments, participated in the design of the study and drafted the manuscript; CD carried out the statistical analyses; EF collected field data; GH conceived of the study, designed the study, coordinated the study and helped draft the manuscript. All authors gave final approval for publication.

• Acknowledgements
Please acknowledge anyone who contributed to the study but did not meet the authorship criteria.

• Funding statement
Please list the source of funding for each author.

Once again, thank you for submitting your manuscript to Royal Society Open Science and I look forward to receiving your revision. If you have any questions at all, please do not hesitate to get in touch.

Kind regards,
Andrew Dunn
Royal Society Open Science Editorial Office
Royal Society Open Science
openscience@royalsociety.org

on behalf of Professor Rachel Wood (Associate Editor) and Kevin Padian (Subject Editor)
openscience@royalsociety.org

Associate Editor's comments (Professor Rachel Wood):

Both reviewers agree that this is an account of a potentially interesting new appendage, but the case for this being a radiodont appendage needs to be strengthened, and also please offer further evidence to support the proposition that this animal was a filter feeder.

Comments to Author:

Reviewers' Comments to Author:
Reviewer: 1

Comments to the Author(s)
The subject of this paper is a single small, fragmentary fossil preserved as a black film. It is either a hurdiid as the authors argue or else it is a fragment of some other kind of organism. Unlike Ursalinacaris (or other hurdiids), the inferred ventral spines greatly increase in length along a series of inferred proximal to distal podomeres, being really long on inferred podomeres 5 and 6. This could simply be taxonomic novelty (a new way of being a hurdiid) but it is so fundamentally different from radiodont ventral spine variation that it makes me worry that the specimen could be something else completely. Likewise having a very elongate, straight, needle-like terminal spine is off the scale for hurdiids (or radiodonts more widely). The "dorsal spines" are poorly preserved: if it's a radiodont frontal appendage then that is what they are but on their own they're not a strong argument for this being a radiodont. The "auxiliary spines" seem to be OK (they're on the inferred distal edge of the ventral spines and have consistent orientations among the few preserved on the specimen – although, as the authors note, their obliqueness is unlike hurdiids).

The "Adaptability of Radiodonta" section could be shortened. It comes across as trying to extract as much impact as is possible from a single fragment. The major novel conclusions of the paper (such as a radiodont being much smaller than thought possible, and occurring in a setting where radiodonts don't usually seem to have lived) could also be seen as reasons to wonder if it actually is a radiodont.

I can appreciate that it won't be easy to tease much more out of the specimen, e.g., trying different imaging approaches likely won't make it reveal unambiguous characters. But I'd urge the authors to throw everything possible at it.

Reviewer: 2

Comments to the Author(s)
This is a nice account of an interesting new appendage, probably belonging to a radiodont panarthropod. This group is uncommon in post-Cambrian deposits and as such the discovery is of interest for understanding their range and diversity.
I think that the argument that this is a radiodont appendage is reasonably convincing, although it might be strengthened if specific descriptive comparisons were made with other possible identities, for example eurypterids which can sometimes have quite hurdiid-like appendages.
My main concern is that insufficient evidence and argumentation is provided to suggest that this animal was a filter feeder. I find this interpretation unconvincing given the few and very short auxiliary spines visible in the single specimen. Undoubtedly prey size must have been small given the size of the animal, but could it not also have been feeding on small epibenthic or infaunal organisms? I suggest either a more pluralistic discussion of the evidence for and against different feeding types, or to simply acknowledge the uncertainty in interpretation given the limited fossil material.
Specific comments:
45 – "vast" may be an overstatement
134, 175 – Where was this measurement taken? There seem to be very few auxiliary spines and they are sparsely distributed. Are you inferring that some are not visible or not preserved, such that originally there would have been a more continuous array?
160 – Less than 8mm seems like a low estimate for an appendage of length 1.8mm. The reference cited (39) for estimating the size of the complete animal is not suited for this purpose considering that Pahvantia is not known from complete specimens. Refs (7), (26)-(28) include more complete hurdiid specimens for size estimation. Based on a rough estimate from the most complete specimens in these papers, I would think a body size of 10-20mm would be more reasonable. This would make it roughly on par with the small Lyrarapax specimen described in (20) – allowing for some uncertainty in size estimation might warrant tempering claims that this is the smallest known radiodont.
167 – Might be worth emphasizing the caveat that very little is known about early radiodont development (even the smallest juvenile Lyrarapax might be far along in development, if for example it was preceded by a larval stage).
180 – (Also other places where this is discussed in the text) I don't think it's justified to infer convergence without providing a phylogenetic tree in support.
203 – Couldn't the lack of soft/lightly sclerotized organisms be due to taphonomic bias? The general rarity of euarthropods in this biota contrasts with the localized preservation of this radiodont appendage along with a mollisoniid. Could this be the result of atypical local taphonomic conditions?
225 – So you argue that oxygen and water depth are not likely to explain faunal differences. Then you generally invoke regional ecological shifts as an alternative explanation. Is the next section meant to expand on this by proposing that the greater beta diversity in the Ordovician explains the Afon Gam biotic composition? I don't quite see the logic here, as the GOBE may explain the global pattern, but we still lack a cause for the differentiation of the Afon Gam biota from

contemporaneous deposits (be that oxygen, water depth or some other factor not considered). In other words, it may fit the global pattern, but doesn't explain the specific case.
230-245 – the discussion in this section reads a bit disjointed from the rest of the text and the connection with the new fossil material is not very evident. It also seems to mix together a few distinct ideas. I recommend to simplify and clarify this section.

Author's Response to Decision Letter for (RSOS-200459.R0)

See Appendix A.

Decision letter (RSOS-200459.R1)

We hope you are keeping well at this difficult and unusual time. We continue to value your support of the journal in these challenging circumstances. If Royal Society Open Science can assist you at all, please don't hesitate to let us know at the email address below.

Dear Dr Pates,

It is a pleasure to accept your manuscript entitled "A miniature Ordovician hurdiid from Wales demonstrates the adaptability of Radiodonta" in its current form for publication in Royal Society Open Science. The comments of the reviewer(s) who reviewed your manuscript are included at the foot of this letter.

Please ensure that you send to the editorial office an editable version of your accepted manuscript, and individual files for each figure and table included in your manuscript. You can send these in a zip folder if more convenient. Failure to provide these files may delay the processing of your proof. You may disregard this request if you have already provided these files to the editorial office.

You can expect to receive a proof of your article in the near future. Please contact the editorial office (openscience_proofs@royalsociety.org) and the production office (openscience@royalsociety.org) to let us know if you are likely to be away from e-mail contact -- if you are going to be away, please nominate a co-author (if available) to manage the proofing process, and ensure they are copied into your email to the journal.

Due to rapid publication and an extremely tight schedule, if comments are not received, your paper may experience a delay in publication.

Please see the Royal Society Publishing guidance on how you may share your accepted author manuscript at https://royalsociety.org/journals/ethics-policies/media-embargo/.

Thank you for your fine contribution. On behalf of the Editors of Royal Society Open Science, we look forward to your continued contributions to the Journal.

Kind regards,
Anita Kristiansen
Editorial Coordinator

Royal Society Open Science
openscience@royalsociety.org

on behalf of Professor Rachel Wood (Associate Editor) and Kevin Padian (Subject Editor)
openscience@royalsociety.org

Follow Royal Society Publishing on Twitter: @RSocPublishing
Follow Royal Society Publishing on Facebook:
https://www.facebook.com/RoyalSocietyPublishing.FanPage/
Read Royal Society Publishing's blog: https://blogs.royalsociety.org/publishing/

Appendix A

Department of Organismic and Evolutionary Biology
26 Oxford Street
Cambridge
MA 02138, USA

15 May 2020

Dear Prof. Wood,

Many thanks for your comments and for organising the review process for this submission. We respond to the points below, with the original comments in bold type and our responses in red type. We thank both reviewers and yourself for the constructive comments on the manuscript. We have taken into consideration all the points raised and, with only a few exceptions, have made improvements to the text based on these comments. The manuscript has been significantly lengthened, and to support the strengthened arguments it contains, we include an additional two figures. We hope you will agree that the issues raised by the reviewers have been addressed and that the manuscript is ready for acceptance.

Thank you for your hard work, and very fast turnaround, especially given the current pandemic. All the best,

Stephen Pates, on behalf of all authors

Associate Editor's comments (Professor Rachel Wood):

Both reviewers agree that this is an account of a potentially interesting new appendage, but the case for this being a radiodont appendage needs to be strengthened, and also please offer further evidence to support the proposition that this animal was a filter feeder.

To strengthen the case for this specimen being a radiodont frontal appendage, we have added in more details concerning key characters for identifying radiodont frontal appendages, and specifically hurdiid appendages, the family to which we propose assigning this specimen. In addition, following the advice of both reviewers, we include comparisons of the material with other groups (lobopodians and eurypterids) in order to strengthen our taxonomic assignment. We agree with Reviewer 2, that from a single partial specimen we should not make too much of the feeding mode for the animal, and so have decided to remove our suggestion that this animal may have been a filter feeder.

We feel that this manuscript has been significantly helped by the peer review process, and thank the editor and the reviewers for their time. We provide point-by-point responses to the reviewer comments below.

Reviewer: 1

The subject of this paper is a single small, fragmentary fossil preserved as a black film. It is either a hurdiid as the authors argue or else it is a fragment of some other kind of organism.

We thank the Reviewer for their comments on the manuscript, which have highlighted areas where we needed to improve the clarity of the message, as well as where the discussion required expansion. Following this comment (and a comment from Reviewer 2) we have added a subsection on alternative taxonomic hypotheses for this specimen. We now briefly discuss why we do not think it is a lobopodian body (as other radiodonts have been misidentified in this way in the past) and also in detail why we do not think it is a eurypterid prosomal appendage (in light of a specific suggestion by Reviewer 2).

Unlike Ursalinacaris (or other hurdiids), the inferred ventral spines greatly increase in length along a series of inferred proximal to distal podomeres, being really long on inferred podomeres 5 and 6.

This comment comes from a misunderstanding of the description, which we have now clarified. The endites on podomeres 2, 3 and 4 are incomplete, and it is inferred that they are no shorter than those on podomeres 5 and 6. A shorter endite on podomere 7 is compatible with a number of hurdiids, and this is now made more explicit as well. A new Figure 4b, showing our reconstruction of the appendage, also serves to make it clear that the endites on podomeres 2-4 are not complete, and that we consider them to have been a similar size to those on podomeres 5 and 6.

This could simply be taxonomic novelty (a new way of being a hurdiid) but it is so fundamentally different from radiodont ventral spine variation that it makes me worry that the specimen could be something else completely. Likewise having a very elongate, straight, needle-like terminal spine is off the scale for hurdiids (or radiodonts more widely).

As mentioned above, the variation in the length of the endites observed by the Reviewer is taphonomic rather than biological. The elongate terminal spine is novel among described hurdiids, however given the other morphological similarities discussed, we feel that this specimen is still best considered as a hurdiid radiodont. If the Reviewer has any additional taxonomic hypotheses they would like us to consider (beyond lobopodian, and the eurypterids discussed) we are happy to include and discuss them.

The “dorsal spines” are poorly preserved: if it’s a radiodont frontal appendage then that is what they are but on their own they’re not a strong argument for this being a radiodont. The “auxiliary spines” seem to be OK (they’re on the inferred distal edge of the ventral spines and have consistent orientations among the few preserved on the specimen – although, as the authors note, their obliqueness is unlike hurdiids).

The obliqueness of the auxiliary spines is unique among hurdiids, but likely represents the plesiomorphic state for radiodonts. We have expanded the discussion of this feature in the manuscript.

The “Adaptability of Radiodonta” section could be shortened. It comes across as trying to extract as much impact as is possible from a single fragment. The major novel conclusions of the paper (such as a radiodont being much smaller than thought

possible, and occurring in a setting where radiodonts don’t usually seem to have lived) could also be seen as reasons to wonder if it actually is a radiodont.

We agree with Reviewer 2, that as post-Cambrian radiodonts are rare, a major and novel finding from this study is an Ordovician member of the group. We have added emphasis to this point at the start of the discussion. However, this also necessitates the discussion of the size of the animal, and the depositional setting where the specimen was discovered, as it differs from Cambrian examples and is unusual for the group. It would be remiss of us not to discuss these topics. We do not agree that size or ecological setting are reasons to discard an interpretation for this animal as a radiodont, and maintain that this taxonomic hypothesis best fits the data available.

I can appreciate that it won’t be easy to tease much more out of the specimen, e.g., trying different imaging approaches likely won’t make it reveal unambiguous characters. But I'd urge the authors to throw everything possible at it.

A number of different imaging techniques were applied to this specimen – photography wet and dry, and using polarized lighting. We do not think that other approaches (e.g. CT scanning) will be successful given the preservation of the material as a black film with no significant density difference expected.

Reviewer: 2

This is a nice account of an interesting new appendage, probably belonging to a radiodont panarthropod. This group is uncommon in post-Cambrian deposits and as such the discovery is of interest for understanding their range and diversity.
We thank the Reviewer for their positive comments regarding the manuscript, and constructive comments below. We have made changes relating to all of them (with one exception) and feel that they have helped to significantly improve the manuscript.

I think that the argument that this is a radiodont appendage is reasonably convincing, although it might be strengthened if specific descriptive comparisons were made with other possible identities, for example eurypterids which can sometimes have quite hurdiid-like appendages.
We have added a subsection comparing this specimen to some eurypterids as suggested by the Reviewer, in addition to a new figure (Figure 5) comparing Ordovician spiniferous appendage types known, and one Devonian sweep-feeding appendage which bears spines of a similar morphology to what is seen in the Welsh specimen considered in this study. If there are other specific eurypterid taxa that we have overlooked and not considered in this comparison, we would be very happy to include them.

My main concern is that insufficient evidence and argumentation is provided to suggest that this animal was a filter feeder. I find this interpretation unconvincing given the few and very short auxiliary spines visible in the single specimen. Undoubtedly prey size must have been small given the size of the animal, but could it not also have been feeding on small epibenthic or infaunal organisms? I suggest either a more pluralistic discussion of the evidence for and against different feeding types, or to simply acknowledge the uncertainty in interpretation given the limited fossil material.
We agree with the reviewer that we overstretched this point in the original submission, and have removed any mention of this animal being a filter feeder. In addition, we acknowledge the uncertainty in the presence of auxiliary spines along the distal margin of the endite (as we originally inferred).

Specific comments:

45 – “vast” may be an overstatement
This term has been deleted.

134, 175 – Where was this measurement taken? There seem to be very few auxiliary spines and they are sparsely distributed. Are you inferring that some are not visible or not preserved, such that originally there would have been a more continuous array?
The measurement was taken between the two spines with black arrows indicated in Figure 3c. This has now been specified in the text, and a short discussion of whether there was a more continuous array or not along the endites has been added. The reconstruction (Figure 4b) will also help to remove uncertainty as to what features have been inferred.

160 – Less than 8mm seems like a low estimate for an appendage of length 1.8mm. The reference cited (39) for estimating the size of the complete animal is not suited for this purpose considering that Pahvantia is not known from complete specimens. Refs (7), (26)-(28) include more complete hurdiid specimens for size estimation. Based on a

rough estimate from the most complete specimens in these papers, I would think a body size of 10-20mm would be more reasonable. This would make it roughly on par with the small Lyrarapax specimen described in (20) – allowing for some uncertainty in size estimation might warrant tempering claims that this is the smallest known radiodont.
This section exploring the extrapolated size of the adult of this specimen has been significantly expanded following this comment. It is necessary to include a specific Reference [39], as this study compiled data relating the lengths of frontal appendages to body length in all radiodonts (not just *Pahvantia*) alongside data on central carapace element length to body length. We have added references to the primary literature in addition to this Reference. Data from *Peytoia nathorsti* supports an extrapolated body size of under 10 mm. For *Hurdia* and *Cambroraster* (References [26-28] suggested by the reviewer) using frontal appendages to extrapolate to the specimen length is more complicated. In [39] the carapace length of *Hurdia* was used rather than the frontal appendage for this reason (as well as to facilitate comparison with carapaces of *Pahvantia*). An attempt has been made to use the frontal appendages of these two animals following the Reviewer's comment, and a range (rather than a single value) is now used. The uppermost bound (15 mm) comes from *Cambroraster*, but this is not a strong extrapolation as it involves a two-step calculation. For *Hurdia* one specimen in ventral view shows the relative length of the appendages to the body, although the appendage is not complete (as it is slightly overlain by the oral cone in this view). This specimen is broadly consistent with *Peytoia* however we acknowledge that other specimens of *Hurdia* with incomplete appendages may indicate a slightly different ratio closer to but not exceeding what is calculated for *Cambroraster*.

167 – Might be worth emphasizing the caveat that very little is known about early radiodont development (even the smallest juvenile Lyrarapax might be far along in development, if for example it was preceded by a larval stage).
We have added a short section briefly discussing the limits of what is known about radiodont development, and the possibility of a larval stage.

180 – (Also other places where this is discussed in the text) I don't think it's justified to infer convergence without providing a phylogenetic tree in support.
We have removed any suggestion that this animal was involved in filter-feeding, and also all instances where convergence was considered.

203 – Couldn't the lack of soft/lightly sclerotized organisms be due to taphonomic bias? The general rarity of euarthropods in this biota contrasts with the localized preservation of this radiodont appendage along with a mollisoniid. Could this be the result of atypical local taphonomic conditions?
This question (whether the lack of euarthropods is due to taphonomic bias) was considered in the original description of the biota (Reference [49]), where it was concluded that the rarity of euarthropods was not taphonomic, but instead reflects a true ecological signal. A reference to this study has been included to support the statement within the manuscript.

225 – So you argue that oxygen and water depth are not likely to explain faunal differences. Then you generally invoke regional ecological shifts as an alternative explanation. Is the next section meant to expand on this by proposing that the greater beta diversity in the Ordovician explains the Afon Gam biotic composition? I don't quite see the logic here, as the GOBE may explain the global pattern, but we still lack a

cause for the differentiation of the Afon Gam biota from contemporaneous deposits (be that oxygen, water depth or some other factor not considered). In other words, it may fit the global pattern, but doesn't explain the specific case.
The argument in this section is that common explanations for differences in the biota (oxygen and water depth) cannot be applied to explain the differences in the Afon Gam Biota compared to other radiodont-hosting faunas. It has been observed that Ordovician communities are more different from each other than Cambrian communities are from each other. Each individual Ordovician community, of which the Afon Gam biota is one, presents different ecological conditions. The small size of the Welsh hurdiid is proposed as one way in which radiodonts have shown themselves to be able to adapt to different ecological environments – here one in which motile food sources are rare. We have added a short sentence to address the specific case of the Afon Gam Biota at the end of this section, and to bring the argument full circle.

230-245 – the discussion in this section reads a bit disjointed from the rest of the text and the connection with the new fossil material is not very evident. It also seems to mix together a few distinct ideas. I recommend to simplify and clarify this section.
We have rewritten this section completely, to summarize the findings more clearly.
